# MicroRNAs 21 and 199a-3p Regulate Axon Growth Potential through Modulation of *Pten* and *mTo*r mRNAs

**DOI:** 10.1523/ENEURO.0155-21.2021

**Published:** 2021-08-10

**Authors:** Amar N. Kar, Seung-Joon Lee, Pabitra K. Sahoo, Elizabeth Thames, Soonmoon Yoo, John D. Houle, Jeffery L. Twiss

**Affiliations:** 1 Department Biological Sciences, University of South Carolina, Columbia, SC 29208; 2 Nemours Biomedical Research, Alfred I. duPont Hospital for Children, Wilmington, DE 19803; 3 Department of Neurobiology and Anatomy, Drexel University College of Medicine, Philadelphia, PA 19129

**Keywords:** axonal mRNA, axonal translation, microRNA, PTEN, regeneration, translation

## Abstract

Increased mTOR activity has been shown to enhance regeneration of injured axons by increasing neuronal protein synthesis, while PTEN signaling can block mTOR activity to attenuate protein synthesis. MicroRNAs (miRs) have been implicated in regulation of PTEN and mTOR expression, and previous work in spinal cord showed an increase in miR-199a-3p after spinal cord injury (SCI) and increase in miR-21 in SCI animals that had undergone exercise. Pten mRNA is a target for miR-21 and miR-199a-3p is predicted to target mTor mRNA. Here, we show that miR-21 and miR-199a-3p are expressed in adult dorsal root ganglion (DRG) neurons, and we used culture preparations to test functions of the rat miRs in adult DRG and embryonic cortical neurons. miR-21 increases and miR-199a-3p decreases in DRG neurons after in vivo axotomy. In both the adult DRG and embryonic cortical neurons, miR-21 promotes and miR-199a-3p attenuates neurite growth. miR-21 directly bound to Pten mRNA and miR-21 overexpression decreased Pten mRNA levels. Conversely, miR-199a-3p directly bound to mTor mRNA and miR-199a-3p overexpression decreased mTor mRNA levels. Overexpressing miR-21 increased both overall and intra-axonal protein synthesis in cultured DRGs, while miR-199a-3p overexpression decreased this protein synthesis. The axon growth phenotypes seen with miR-21 and miR-199a-3p overexpression were reversed by co-transfecting PTEN and mTOR cDNA expression constructs with the predicted 3′ untranslated region (UTR) miR target sequences deleted. Taken together, these studies indicate that injury-induced alterations in miR-21 and miR-199a-3p expression can alter axon growth capacity by changing overall and intra-axonal protein synthesis through regulation of the PTEN/mTOR pathway.

## Visual Abstract

**Figure:**
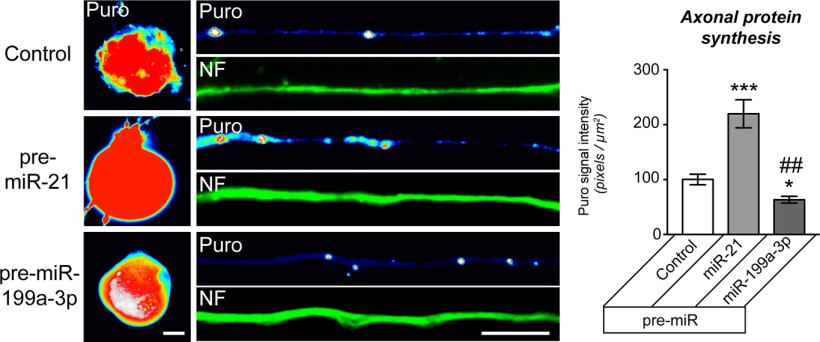

## Significance Statement

Posttranscriptional regulation of neuronal gene expression has been shown to contribute to axon regeneration after nervous system injury. We show that the PTEN/mTOR pathway is regulated by microRNAs (miRs) after neuronal injury and this modifies the capacity for translation of regeneration-associated genes in primary neurons.

## Introduction

Posttranscriptional regulation of gene expression has proven to be a critical mechanism driving neuronal development, function, and injury responses (Popovitchenko and Rasin, 2017; Bae and Miura, 2020; Dalla Costa et al., 2021). These regulatory mechanisms include processing RNA transcription products in the nucleus, stabilizing or destabilizing mRNAs in the cytoplasm, subcellular localization of mRNAs, and translational regulation. Non-coding microRNAs (miRs) contribute to both stability and translational regulation of mRNAs by binding to specific seed sequences in 3′ untranslated regions (UTRs) of cellular mRNAs. Previous work showed that miR-199a-3p, which was predicted to target *mTOR* mRNA, is upregulated in spinal cord after a complete transection spinal cord injury (SCI) at thoracic level 10 (T10; Liu et al., 2012). Exercise after SCI has been shown to alter gene expression leading to increased spinal cord plasticity and recovery of spinal reflexes (Mendell et al., 2001; Ying et al., 2005; Côté et al., 2011), and passive hindlimb exercise after SCI was shown to attenuate the SCI-induced increase in miR-199a-3p with a concurrent upregulation of miR-21 (Liu et al., 2012). miR-21 has been shown to target *Pten* mRNA (Xu et al., 2019; Lopez-Leal et al., 2020) and miR199a-3p is predicted to target *mTor* mRNA (Liu et al., 2012). PTEN has been implicated in blocking axon regrowth in both the central and peripheral nervous systems (Park et al., 2008; Abe et al., 2010; Christie et al., 2010; Liu et al., 2010). Among the downstream effects of PTEN signaling is attenuation of mTOR activity to decrease mRNA translation (Switon et al., 2017). Thus, it is intriguing to speculate that the changes in miR-199a-3p and miR-21 reported after SCI and SCI plus passive hindlimb exercise (Liu et al., 2012) might alter the growth potential of injured neurons. Here, we have asked whether miR-21 and miR-199a-3p are expressed in adult neurons and the mechanisms underlying their effects in neurons.

Adult dorsal root ganglion (DRG) neurons extend axonal projections in culture in the absence of exogenous growth factors and this growth can be increased by a preconditioning injury (Smith and Skene, 1997) and by voluntary exercise (Molteni et al., 2004). These preconditioned neurons rapidly extend axons through translational regulation of mRNAs rather than new gene transcription during the culture period (Twiss et al., 2000). Sciatic nerve injury also preconditions DRG neurons for regeneration after subsequent SCI (Neumann and Woolf, 1999). Here, we confirm that miR-21 promotes axon growth by depletion of *Pten* mRNA and protein resulting in increased neuronal protein synthesis, and we find that miR-199a-3p attenuates axon growth by depletion of *mTor* mRNA and protein resulting in decreased neuronal protein synthesis. Furthermore, miR-199a-3p localizes to axons where it prevents axonal *mTor* mRNA translation to decrease axon growth and this is overcome by inhibition of miR-199a-3p. Importantly, we provide evidence that overexpression of miR-21 or inhibition of miR-199a-3p allows both DRG and embryonic cortical neurons to extend axons on the non-permissive substrate chondroitin sulfate proteoglycan (CSPG), indicating that these miRs directly impact the neuron’s ability to extend axons over a previously inhospitable terrain.

## Materials and Methods

### Animal care and use

All vertebrate animal experiments were performed under protocols approved by the Institutional Animal Care and Use Committee. Male Sprague Dawley rats (175–250 g) were used for DRG culture and sciatic nerve injury experiments. Isofluorane was used for anesthesia during the peripheral nerve injury procedure. For peripheral nerve injury, anesthetized rats were subjected to a sciatic nerve crush at mid-thigh as described previously (Twiss et al., 2000). Embryonic day (E)18 male and female rat pups were used for cortical neuron cultures. Animals were euthanized using CO_2_ asphyxiation per approved methods.

### Primary neuron culture

For primary DRG cultures, L4-5 DRG were harvested in Hibernate-A medium (BrainBits) and then dissociated with collagenase (Life Technologies). After centrifugation and washing in DMEM/F12 (Life Technologies), cells were resuspended in DMEM/F12, 1 × N1 supplement (Sigma), 10% fetal bovine serum (HyClone), and 10 μm cytosine arabinoside (Sigma). Dissociated DRGs were plated immediately on poly-L-lysine (Sigma) and laminin (Millipore) coated coverslips or transfected and then plated on coated coverslips for staining and imaging or polyethylene-tetrathalate (PET) membrane (1-μm pores; Corning) inserts for isolation of axons. For CSPG experiments, adult DRGs were cultured on glass bottom dishes (Greiner) that were coated with 10 μg/ml Aggrecan (R&D Systems) after the laminin/poly-D-lysine coating above.

For cortical neuron cultures, E18 cortices were dissected in Hibernate E (BrainBits) and dissociated using the Neural Tissue Dissociation kit (Miltenyi Biotec). Dissected cortices were incubated in a prewarmed enzyme mix at 37°C for 15 min; tissues were then triturated and applied to a 40 μm cell strainer. After washing and centrifugation, neurons were seeded onto poly-D-lysine (Sigma) coated coverslips. For the CSPG experiments, cells were seeded on glass bottom dishes (Greiner) coated with 10 μg/ml aggrecan in addition to the poly-D-lysine. NbActive-1 medium (BrainBits) supplemented with 100 U/ml of penicillin-streptomycin (Life Technologies), 2 mm L-glutamine (Life Technologies), and 1 × N21 supplement (R&D Systems) was used as culture medium.

For isolation of axons from cell bodies, the lower membrane surface for DRGs cultures in membrane inserts was carefully scraped as previously described (Zheng et al., 2001). The upper membrane surface was used as a “cell body” compartment. These isolates were processed for RNA preparation as outlined below.

For plasmid transfections, dissociated ganglia were pelleted by centrifugation at 100 × *g* for 5 min and resuspended in “nucleofector solution” (Rat Neuron Nucleofector kit; Lonza); 2–5 μg plasmid was electroporated using an AMAXA Nucleofector apparatus (program SCN-8; Lonza). For overexpression or inhibition of miRs, 50 nm of precursor-miRNA (pre-miR) or inhibitor anti-miR were transfected using DharmaFECT 3 reagent (Dharmacon). Scrambled precursor and anti-miR oligomers were used as negative controls. RT droplet digital PCR (ddPCR) and immunoblotting were used to test levels of the endogenous miRNA targets (see below).

### DNA constructs

Pre-miRNA, biotinylated miRs, inhibitor anti-miR, and control RNA oligomers were purchased from Exiqon. The myc-tagged mTOR (wild-type) and kinase dead mTOR mutant (mTOR-Kdm) expression constructs were purchased from Addgene (plasmids #1861 and #8482, respectively). All fluorescent reporter constructs for analyses of RNA translation were based on mCherry plasmid with myristoylation element (mCh^MYR^; Vuppalanchi et al., 2012). Reporter constructs containing 5′ and 3′ UTRs of rat *Nrn1*, *Gap4*3, and *Kpnb1* mRNAs have been published (mCh^MYR^5’/3’nrn1 or mCh^MYR^5’/3’kpnb1, or mCh^MYR^5’/3’gap43; Sahoo et al., 2018). For mTOR, the rat 3′ UTR was subcloned by PCR from previously published UTR constructs (Terenzio et al., 2018), and inserted directly downstream of the stop codon of mCh^MYR^ sequence in a vector that included 5′ UTR of rat *CamkII* mRNA (mCh^MYR^5’camKII/3’mtor; Vuppalanchi et al., 2012). Rat PTEN protein coding sequence was generated from a rat DRG cDNA library. Using PCR primers with restriction enzyme sites (EcoRI and SalI), PTEN coding sequence was amplified with high fidelity polymerase (Primer Star HS, Takara) and inserted into pFLAG-CMV2 (Sigma) using EcoRI/SalI sites. All UTR reporter and cDNA expression constructs were confirmed by sequencing.

### RNA isolation and PCR analyses

RNA was isolated from immunoprecipitates and cultures using the RNeasy Micro isolation kit (QIAGEN). Fluorimetry with Ribogreen (Invitrogen) was used to determine RNA quantities. For analyses of total RNA levels and inputs for miR-mRNA co-precipitation analyses, RNA yields were normalized across samples before reverse transcription using Sensifast (Bioline). For quantitating mRNAs co-precipitating with biotinylated miRs (see below), equal proportions of each precipitation were used for reverse transcription with Sensifast. RT products were processed for ddPCR using Evagreen (Bio-Rad) with transcript specific primers (Integrated DNA Tech; sequences available on request). ddPCR reactions were read on a QX200 droplet reader (Bio-Rad).

For miR quantification, tissues and cultures were lysed and total RNA was isolated using the miRVana miRNA Isolation kit (QIAGEN) according to manufacturer’s recommendations. Ribogreen assay was performed to determine the RNA quantity in each sample. Equal amounts of total RNA were reverse transcribed using the miRCURY LNA miRNA RT kit (QIAGEN) according to manufacturer’s protocol. After RT, the miRNAs were detected using miR-specific primers (Exiqon) by ddPCR with Evagreen detection reagent (Bio-Rad) and QX200 droplet reader.

### miR-mRNA coprecipitation

Biotinylated miR mimics (100 nm) were transfected into DRG neurons as above; 16–24 h after transfection, cultures were lysed in 20 mm Tris-HCl (pH 7.5), 10 mm KCl, 5 mm MgCl_2_, and 0.3% NP-40 supplemented with protease and RNase inhibitors. Lysates were cleared by centrifugation for 10 min at 4°C, 18,000 × *g*. Supernatants were collected, and equal volume of the supernatant were incubated with preequilibrated Streptavidin M280 Magnetic Beads (Life Technologies) overnight an end-over-end rotator at 4°C; 10% of the lysate was processed for RNA purification using the miRVana miRNA isolation kit, as described earlier for input. After incubation, the beads were washed in lysis buffer and then processed for RNA isolation as outlined above.

### Immunoblotting

Adult rat DRG cultures (3 d *in vitro*, ∼60,000 neurons/well) were lysed in 50 mm Tris-HCl, 10 mm EDTA, 0.1 mm DTT, 1% SDS, and 1× Mini EDTA-free Proteinase Inhibitor (Roche) 36 h after transfections. Samples were denatured by boiling at 95°C for 5 min. Lysates were cleared of debris by centrifugation at 15,000 × *g* for 15 min at 4°C and then normalized for protein content using BCA assay (Bio-Rad). Normalized protein lysates were fractionated on 8% SDS/PAGE gels and transferred to PVDF membranes (GE Healthcare). After blocking in 5% non-fat dried milk (Bio-Rad) diluted in Tris-buffered saline with 1% Tween 20 (TBST), membranes were probed overnight at 4°C with rabbit anti-mTOR (1:1000; Abcam), rabbit anti-PTEN (1:1000, Abcam), or rabbit anti-GAPDH (1:2000; Cell Signaling Technology) antibodies diluted in blocking buffer. Blots were washed in TBST and then incubated with horseradish peroxidase (HRP)-conjugated anti-rabbit IgG (1:2000; Cell Signaling Technology) diluted in blocking buffer for 1 h at room temperature. Blots were washed in TBST and signals detected with ECL Prime (GE Healthcare).

### Puromycinylation assay

To visualize newly synthesized proteins in cultured DRG neurons, we used the Click-iT Plus OPP Protein Synthesis Assay kit per manufacturer’s instructions (Invitrogen). Briefly, 3 d DRG cultures were incubated with 20 μm O-propargyl-puromycin (OPP) for 30 min at 37°C. OPP-labeled proteins were detected by crosslinking with Alexa Fluor 594 picolyl azide molecule. Coverslips were then mounted with Prolong Gold Antifade (Invitrogen) and imaged with Leica DMI6000 epifluorescent microscope as above. ImageJ (National Institutes of Health) was used to quantify the incorporated puromycinylation signals in distal axons and cell bodies.

### Immunofluorescence

All steps occurred at room temperature unless specified otherwise. DRG and cortical neuron cultures were rinsed in PBS and then fixed in 4% paraformaldehyde in PBS for 20 min. Coverslips were then rinsed in PBS and then permeabilized in PBS plus 0.5% Tween 20. After rinsing in PBS again, coverslips were blocked in 5% donkey serum in PBS plus 0.1% Tween 20 (PBST) for 1 h followed by overnight incubation at 4°C in primary antibodies diluted in blocking buffer. Mouse RT-97 anti-neurofilament (NF; 1:2000; Development Hybridoma Studies Bank) and mouse anti-FLAG (1:2000; Sigma) were used for primary antibodies. Coverslips were rinsed in PBS and then incubated with secondary antibodies diluted in blocking buffer for 1 h. Secondary antibodies consisted of FITC-conjugated goat-anti-mouse IgG antibody and CY5 conjugated anti-rabbit (both at 1:400; Jackson ImmunoResearch). After rinsing in PBS followed by distilled H_2_O, samples were mounted with Prolong Gold antifade.

### Axon growth analyses

For neurite and axon outgrowth, images from 48- to 60-h DRG or cortical neuron cultures were acquired using the ImageXpress Micro High-Content Imaging System (Molecular devices). These images were analyzed for neurite outgrowth using WIS-Neuromath (Rishal et al., 2013). Neurites were visualized using NF immunofluorescence. Neurite lengths were measured for at least 75 neurons per treatment group per culture preparation.

### Fluorescence recovery after photobleaching (FRAP)

FRAP was used to evaluate axonal protein synthesis using diffusion-limited mCherry^MYR^ reporters as described with minor modifications (Yudin et al., 2008). DRG neurons were co-transfected with mCh^MYR^5’camKII/3’mtor, mCh^MYR^5’/3’nrn1, mCh^MYR^5’/3’kpnb1, or mCh^MYR^5’/3’gap43 plus pre-miR-199a-3p or control RNA. Cells were maintained at 37°C, 5% CO_2_ during imaging sequences. 514 nm laser line on Leica SP8X confocal microscope was used to bleach mCh^MYR^ signals (Argon laser at 70% power, pulsed every 0.82 s for 80 frames). Pinhole was set to 3 airy units to ensure full thickness bleaching and acquisition (63×/1.4 NA oil immersion objective). Before photobleaching, neurons were imaged every 60 s for 2 min to acquire baseline fluorescence for the region of interest (ROI). The same excitation and emission parameters were used to assess recovery over 15 min postbleach with images acquired at 30 s intervals. To determine whether fluorescence recovery in axons because of translation, DRG cultures were treated with 100-μm anisomycin (Sigma) for 30 min before photobleaching. To evaluate whether fluorescence recovery was mTOR-dependent, DRG cultures were treated with 10-nm rapamycin (Sigma) for 30 min before photobleaching.

Fluorescent intensities in the ROIs were calculated using Leica LASX software. For normalizing across experiments, fluorescence intensity value at *t* = 0 min postbleach from each image sequence was set as 0% and prebleach values at 100%. The percentage of fluorescence recovery at each time point after photobleaching was then calculated by normalizing relative to the prebleach fluorescence (Vuppalanchi et al., 2010).

### Statistical analyses

Prism (GraphPad) was used for all statistical analyses. One-way ANOVA with Tukey’s *post hoc* correction was used in all experiments unless specified. For the statistical analyses of FRAP studies two-way ANOVA with Tukey’s *post hoc* test was used; *p* < 0.05 was considered significant. Numbers of experimental replicates are indicated in figure legends.

## Results

### DRG levels of miR-21 and miR-199a-3p change on axotomy

**Figure 1.**
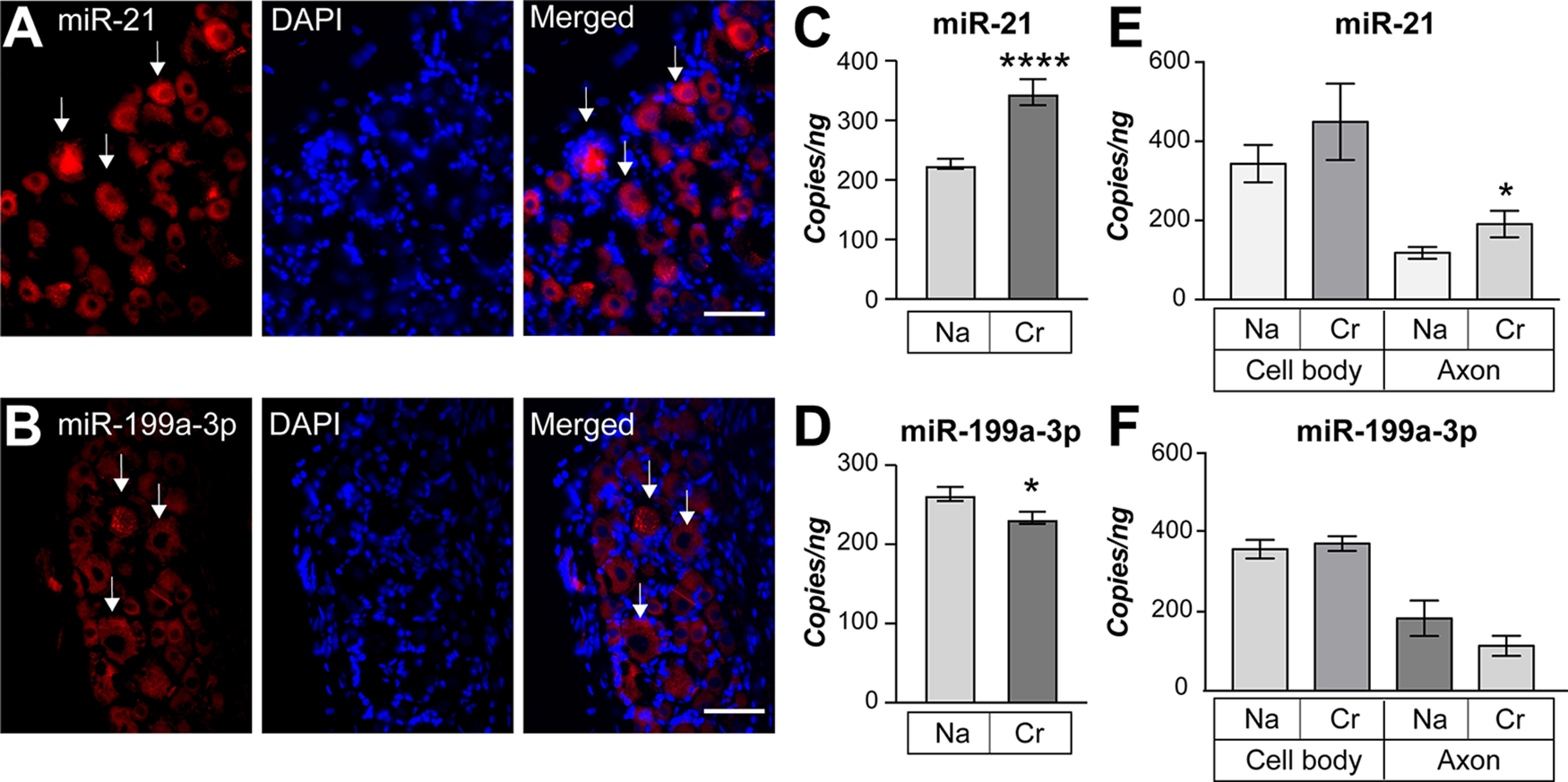
miR-21 and miR-199a-3p are expressed in adult DRG neurons. ***A***, ***B***, Exposure matched images for endogenous miR-21 (***A***) or miR-199a-3p (***B***) plus DAPI in DRG sections derived from adult naive rats. Arrows indicate miR-expressing DRG neurons. Scale bar: 50 μm. ***C***, ***D***, Quantification of mature miR-21 (***C***) and miR-199a-3p (***D***) levels in dissociated DRG neurons derived from naive (Na) and 7-d injury-conditioned (Cr) animals by RT-ddPCR analyses shown as average copies ± SEM per ng of RNA input. ***E***, ***F***, Quantification of miR-21 (***E***) and miR-199a-3p (***F***) levels in cell bodies and axons isolated from naive and injury conditioned DRG neurons is shown as average copies ± SEM per ng of RNA input (*N* ≥ 4 for ***C–F***; *p ≤ 0.05, ****p ≤ 0.001 vs naive axon by Student’s *t* test vs naive axon by Student’s *t* test).

Although spinal cord levels of miR-199a-3p were shown to be increased after SCI and those of miR-21 increased when SCI animals are subjected to exercise (Liu et al., 2012; Li et al., 2020), the cellular source of these miRs was not clear from these previous publications. Moreover, the predictions that miR-21 would target neuronal *Pten* mRNA and miR-199a-3p would target neuronal *mTor* mRNA were not specifically tested. We addressed these knowledge gaps using rat DRG neurons that can be cultured from adult animals and show increased regeneration in the injured spinal cord after SCI when they are preconditioned by a peripheral nerve crush injury (Neumann and Woolf, 1999; Neumann et al., 2002). We initially used fluorescence *in situ* hybridization (FISH) to test for expression of these miRs in the DRGs *in vivo.* With locked nucleic acid (LNA) probes specific for mature miRs, we see clear cytoplasmic signals for both miR-21 and miR-199a-3p in the DRG neurons (Fig. 1*A*,*B*). Unfortunately, the LNA FISH signals were not amenable to quantification, so we used RNA extracted from DRG culture preparations to gain a better understanding of the levels of these miRs in the DRG neurons. DRG neurons that have been preconditioned by an *in vivo* axotomy show rapid axonal outgrowth in culture that is translationally regulated (Smith and Skene, 1997; Twiss et al., 2000). By reverse transcriptase coupled ddPCR (RTddPCR) there was a significant increase in miR-21 and decrease in miR-199a-3p levels in the L4-6 DRGs harvested ipsilateral to a sciatic nerve crush lesion performed 7 d before culture (Fig. 1*C*,*D*).miRs have been shown to localize into sensory axons and their levels can dynamically change during regeneration (Kim et al., 2015; Phay et al., 2015). Since translation of mRNAs in axons has been shown to facilitate regeneration in the PNS (Smith et al., 2020), we asked whether these miRs localize into axons and whether their axonal levels change with the injury conditioning. For this, naive and 7-d injury-conditioned L4-6 dissociated DRGs were cultured on a porous membrane for isolation of axons (Willis and Twiss, 2010). Interestingly, the axon preparations from injury-conditioned DRGs showed significantly increased miR-21 levels (Fig. 1*E*). The axonal levels of miR-199a-3p were decreased, but this did not reach statistical significance (Fig. 1*F*). Neither miR-199a-3p nor miR-21 levels significantly changed in cell body preparations with injury conditioning (Fig. 1*E*,*F*). Thus, consistent with previous observations in mouse DRGs (Cheng et al., 2015), we find that rat DRG neurons express both miR-21 and miR-199a-3p but also that these expression of these miRs changes with injury conditioning and both miRs localize to axons.

### miR-21 and miR-199a-3p target neuronal *Pten* and *mTor* mRNAs

**Figure 2.**
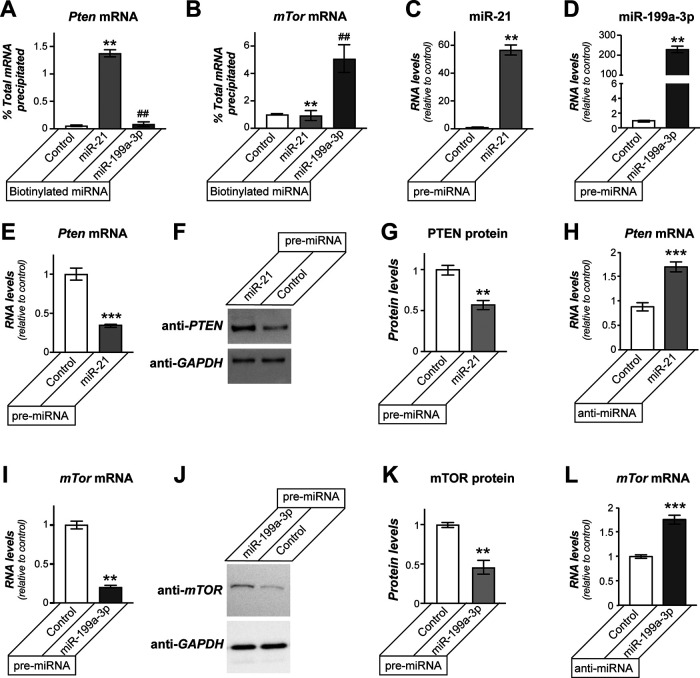
miR-21 and miR-199a-3p target *mTor* and *Pten* mRNAs. ***A***, ***B***, RTddPCR for *Pten* (***A***) and *mTor* (***B***) mRNAs from precipitations of 3’biotinylated miR-21, miR-199a-3p or scrambled RNA (control) transfected DRG neurons is shown as average relative to total input mRNA ± SEM (*N* ≥ 4; **p ≤ 0.01 vs control and ##*p* ≤ 0.01 vs miR-21 by one-way ANOVA with pair-wise comparison and Tukey’s *post hoc* tests). ***C***, ***D***, Transfection of precursor miR-21 and miR-199a-3p (pre-miR-21 and pre-miR-199a-3p, respectively) into DRG neurons increased levels of mature miR-21 (***C***) and miR-199a-3p levels (***D***). ***E–G***, DRG cultures transfected with pre-miR-21 showed decrease in *Pten* mRNA by RTddPCR (***E***) and PTEN protein by immunoblotting (***F***, ***G***). ***F***, Representative immunoblot for PTEN protein, with GAPDH immunoblot showing relatively equal loading between lanes. ***E***, ***K***, Average ± SEM. ***H***, Anti-miR-21 transfection in adult DRG neuron cultures increases levels of *Pten* mRNA. ***I–K*,** DRG cultures transfected with pre-miR-199a-3p showed decrease in *mTor* mRNA by RTddPCR (***I***) and mTOR protein by immunoblotting (***J***, ***K***). ***F***, Representative immunoblot for mTOR protein, with GAPDH immunoblot showing relatively equal loading between lanes. ***I***, ***K***, Average values ± SEM. ***L***, Anti-miR-199a-3p transfection in adult DRG neuron cultures increases levels of *mTor* mRNA (*N* = 4 for ***E***, ***H–J***; *N* = 3 for ***G***, ***K***; **p ≤ 0.01 and ***p ≤ 0.005, vs control by Student’s *t* test).

We next asked whether miR-21 and miR-199a-3p target the predicted *Pten* and *mTor* mRNAs in the adult DRG cultures. For this, we transfected biotinylated miR-21 and miR-199a-3p mimics into naive adult DRG cultures and used RT-ddPCR to determine whether the mRNAs were bound in streptavidin (SA) precipitations. *Pten* mRNA was highly enriched in the miR-21 precipitates as compared with the scrambled control RNA SA precipitations (Fig. 2*A*). In contrast, *mTor* mRNA was highly enriched in the miR-199a-3p compared with scrambled control RNA SA precipitations (Fig. 2*B*). Importantly, *mTor* mRNA was not significantly different from control RNA in the miR-21 precipitates and *Pten* mRNA was not significantly different from control RNA in the miR-199a-3p precipitates (Fig. 2*A*,*B*).

We next transfected DRG cultures with miR precursor RNAs (pre-miRs) to exogenously elevate miR-21 and miR-199a-3p levels versus the corresponding anti-miRs to inhibit functions of endogenous miR-21 and miR-199a-3p in DRG cultures. As expected, transfections with pre-miR-21 and pre-miR-199a-3p significantly increased levels of the mature miR-21 and miR-199a-3p, respectively (Fig. 2*C*,*D*). Pre-miR-21 transfection significantly decreased endogenous *Pten* mRNA and protein levels (Fig. 2*E–G*). Pre-miR-199a-3p transfection significantly decreased endogenous *mTor* mRNA and protein levels (Fig. 2*I–K*). Conversely blocking the functions of endogenous miR-21 using a specific anti-miR increased endogenous *Pten* mRNA levels (Fig. 2*H*), while anti-miR-199a-3p increased endogenous *mTor* mRNA levels (Fig. 2*L*). Taken together, these data point to *Pten* and *mTor* mRNAs as direct targets for miR-21 and miR-199a-3p, respectively, in adult DRG neurons.

### Modulation of miR-21 and miR199a-3p levels alters axon outgrowth in DRG neurons

**Figure 3.**
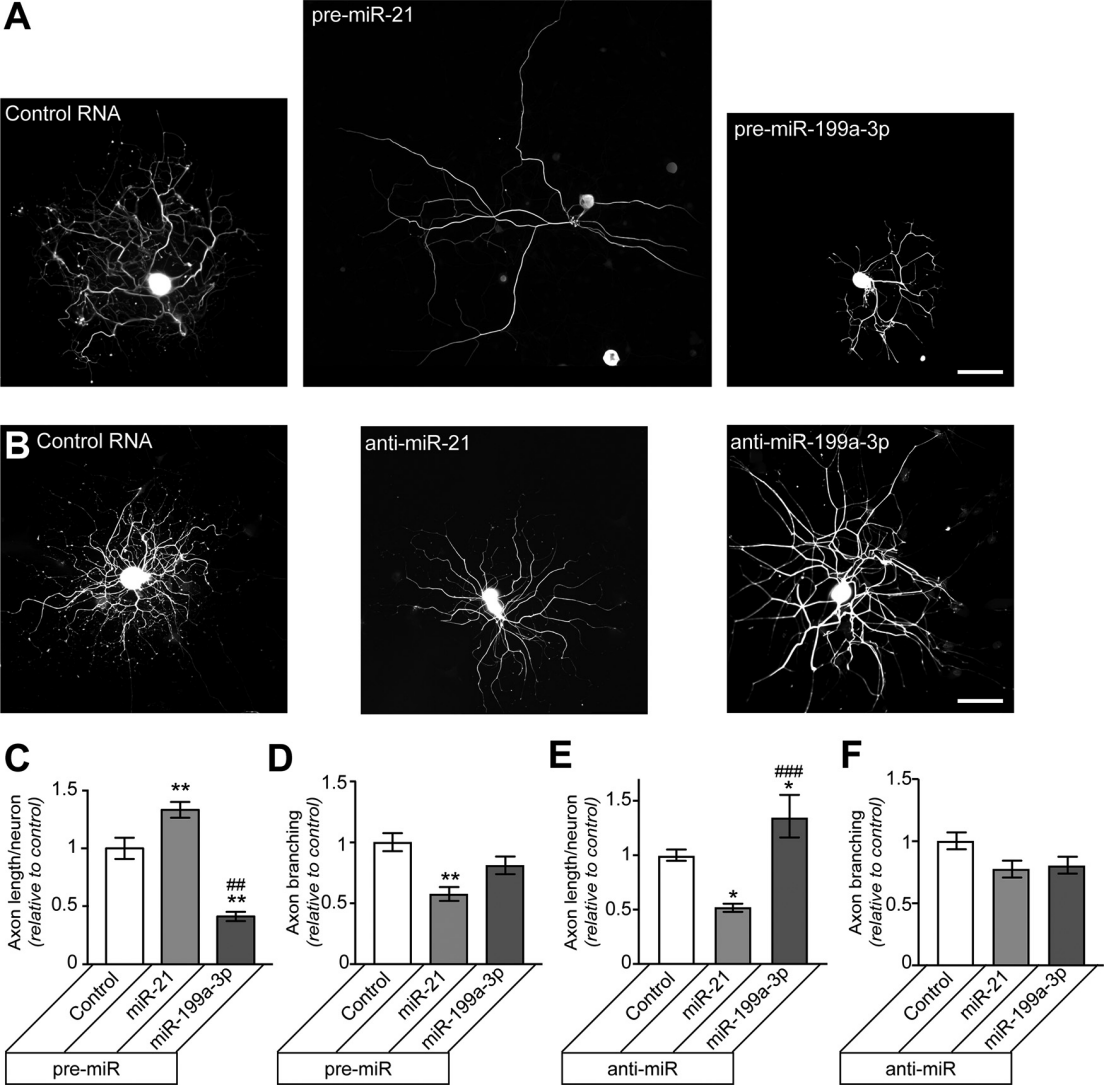
Changing miR-21 and miR-199a-3p levels alters axon growth in DRG neurons. ***A***, ***B***, Representative immunofluorescent images for NF in rat DRG cultures transfected with indicated pre-miRs versus scrambled RNA (control; ***A***) or anti-miRs versus scrambled RNA (***B***) are shown. ***C***, ***D***, Total axon length per neuron (***C***) and axon branching (***D***) are shown for DRG neurons transfected with pre-miRs as in ***A*** as average ± SEM relative to scrambled RNA transfection. ***E***, ***F***, Total axon length per neuron (***E***) and axon branching (***F***) are shown DRG neurons transfected with anti-miRs as in ***B*** as average ± SEM relative to control (*n* ≥ 100 neurons each over 3 experimental replicates for ***C–F***; **p ≤ 0.01 vs control and ##*p* ≤ 0.01, ###*p* ≤ 0.005 comparing between pre-miRs and anti-miRs using one-way ANOVA with pair-wise comparison and Tukey’s *post hoc* tests). Scale bars: 100 μm (***A***, ***B***).

Considering the miR expression and mRNA target studies above together with the reported roles of PTEN to mTOR pathway in axon growth (Park et al., 2008; Abe et al., 2010; Sun et al., 2011; Duan et al., 2015; Chen et al., 2016; Terenzio et al., 2018), we asked whether manipulation of miR-21 and miR-199a-3p levels and function might affect axon growth in the DRG neurons. DRG neurons transfected with pre-miR-21 showed significantly increased axon lengths, while those transfected with pre-miR-199a-3p showed significantly decreased axon lengths (Fig. 3*A*,*C*). The pre-miR-21 transfected neurons also showed significantly decreased axon branching (Fig. 3*A*,*D*). In contrast, DRGs transfected with anti-miR-21 showed reduced axon lengths and those transfected with anti-miR-199a-3p showed increased axon lengths (Fig. 3*B*,*E*). The anti-miR transfections has no significant effect on axon branching (Fig. 3*B*,*F*).

**Figure 4.**
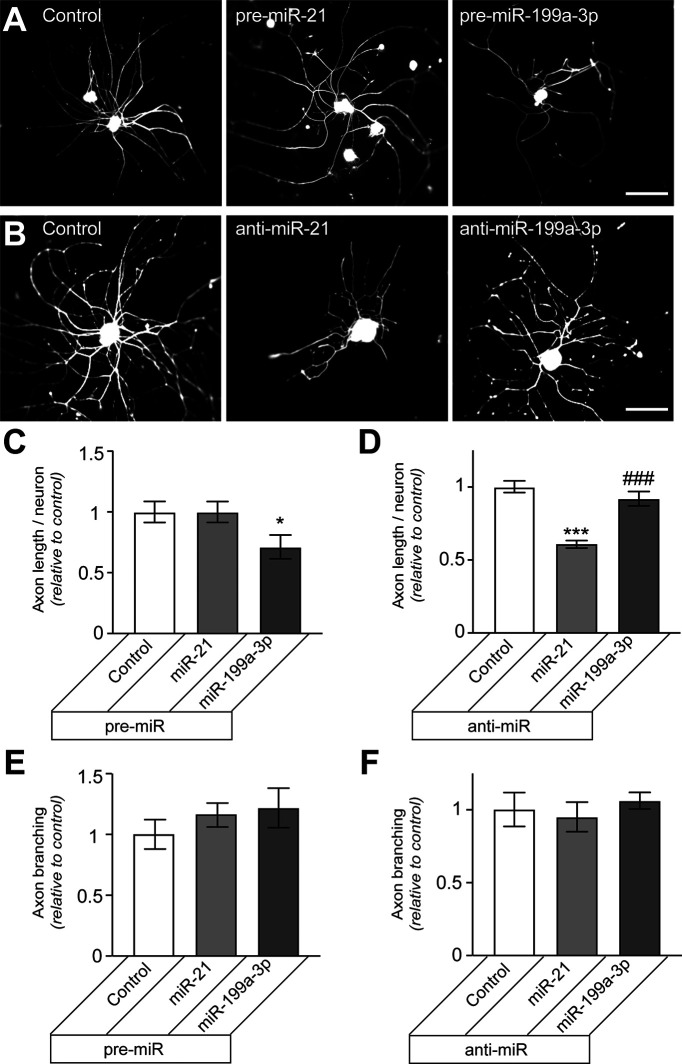
Overexpressing miR-199a-3p or blocking miR-21 decrease axon growth in injury-conditioned DRG neurons. ***A***, ***B***, Representative immunofluorescent images for NF in rat L4-5 DRG neurons that were injury conditioned by sciatic nerve crush 7 d before culture are shown. ***A***, DRGs transfected with indicated pre-miRs versus scrambled RNA (control). ***B***, DRGs transfected with anti-miRs versus scrambled RNA. ***C***, ***D***, Total axon length per neuron is shown for the injury conditioned DRG neurons that were transfected with the indicated pre-miRs (***C***) or anti-miRs (***D***) as average ± SEM relative to control. ***E***, ***F***, Axon branching for injury-conditioned neurons transfected with the indicated pre-miRs (***E***) or anti-miRs (***F***) shown as average ± SEM relative to control (*n* ≥ 150 neurons each over 3 experimental replicates for ***C–F***; *p ≤ 0.05 and ***p ≤ 0.005 vs control and ###*p* ≤ 0.005 comparing between pre-miRs and anti-miRs using one-way ANOVA with pair-wise comparison and Tukey’s *post hoc* tests; no significant differences were for axon branching). Scale bars: 100 μm (***A***, ***B***).

The increased axon length and decreased axon branching seen with miR-21 overexpression is reminiscent of the “elongating” growth morphology that Smith and Skene (1997) described for injury-conditioned DRG neurons (Smith and Skene, 1997). With the elevation of endogenous miR-21 and decrease in endogenous miR-199a-3p in injury-conditioned neurons seen in Figure 1, we asked whether manipulating miR-21 and miR-199a-3p levels and function affects axon growth from injury-conditioned DRG neurons. Seven-day injury-conditioned DRG cultures transfected with pre-miR-21 showed no change in axon length compared with control, but the pre-miR-199a-3p transfected neurons showed significantly decreased axon lengths (Fig. 4*A*,*C*). In contrast, anti-miR-21 transfection significantly decreased axon length in the injury-conditioned DRG neurons, but anti-miR-199a-3p had no effect (Fig. 4*B*,*D*). Neither pre-miRs nor anti-miRs had any significant effect on axon branching (Fig. 4*E*,*F*). Taken together with the miR expression data shown for naive versus injury-conditioned neurons shown in Figure 1*A*, these results point to the increase in miR-21 and decrease in miR-199a-3p seen in the injury-conditioned DRG cultures as drivers for the elongating axon growth from these neurons. Considering that axon growth from injury-conditioned neurons requires translation of an mRNA cohort that exists at the time of culture (Twiss et al., 2000), these data not unsurprisingly raise the possibility that mTOR contributes to the translation of those mRNAs needed for the rapid axon elongation from injury-conditioned neurons.

### Modulation of miR-199a-3p and miR-21 levels alters neurite outgrowth from cortical neurons

**Figure 5.**
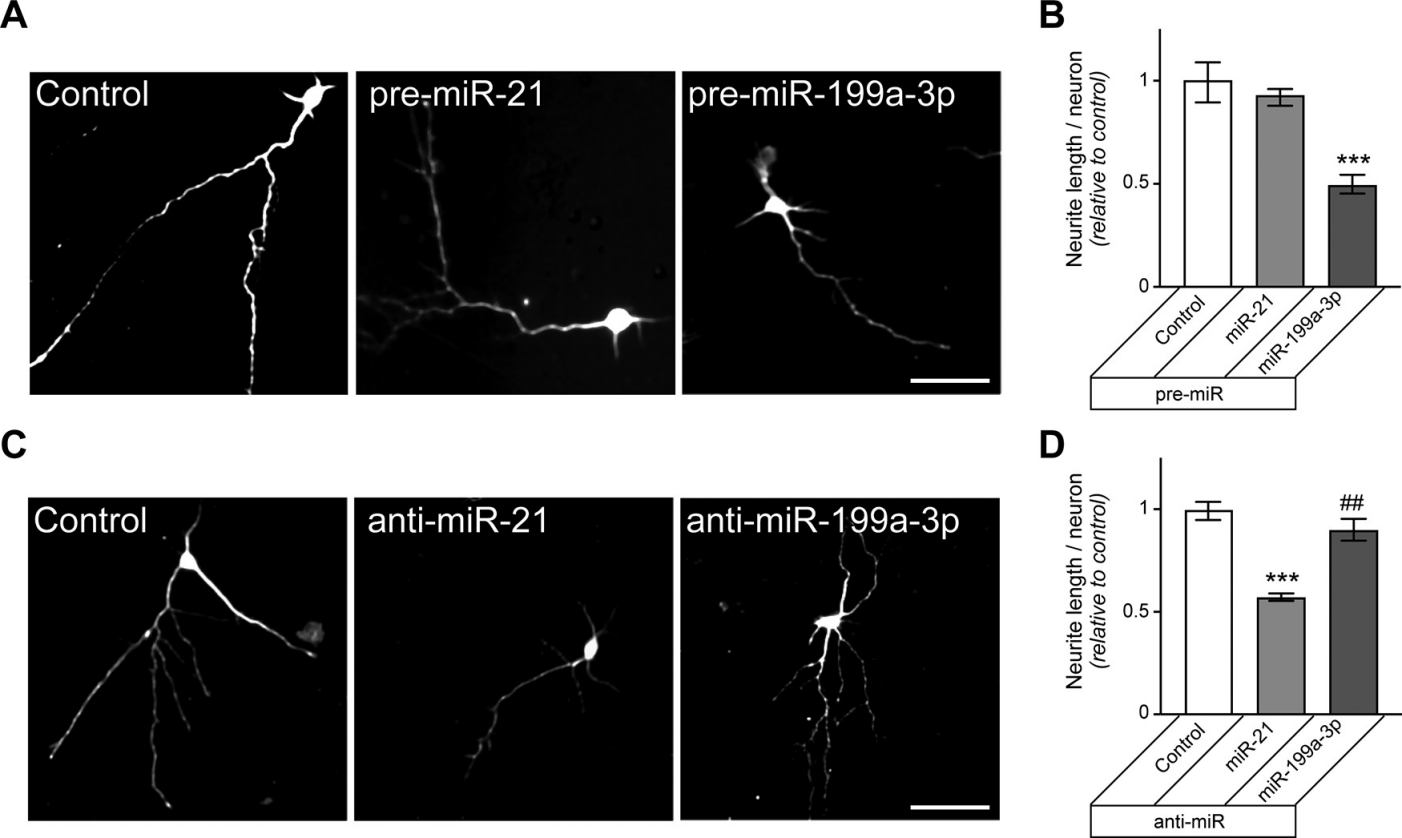
miR-21 and miR-199a-3p levels alter neurite growth in cortical neurons. ***A***, ***B***, Representative immunofluorescent images for NF-stained E18 cortical neurons transfected with indicated pre-miRs versus scrambled RNA (control) are shown in ***A***. Total neurite length per transfected neuron is shown in ***B*** as average ± SEM relative to control (*n* ≥ 95 each over 3 experimental replicates; ***p ≤ 0.0001 vs control and pre-miR-21 by one-way ANOVA with pair-wise comparison and Tukey’s *post hoc* tests). ***C***, ***D***, Representative NF immunofluorescent images for E18 cortical neurons transfected with indicated anti-miRs versus or scrambled RNA are shown in ***C***. Total neurite length per transfected neuron is shown in ***D*** as average ± SEM relative to control (*N* ≥ 95 each over 3 experimental replicates for ***B***, ***D***; ***p ≤ 0.001 vs control and ##*p* ≤ 0.005 vs anti-miR-21 by one-way ANOVA with pair-wise comparison and Tukey’s *post hoc* tests). Scale bars: 50 μm (***A***, ***B***).

Since the PTEN-mTOR pathway has also been shown to affect axon regeneration after optic nerve and corticospinal tract injuries (Park et al., 2008; Liu et al., 2010), we tested whether miR-21 and miR-199a-3p might also affect growth potential of CNS neurons. For this, dissociated E18 cortical neurons were transfected with pre-miRs or anti-miRs at the time of culture and then assessed for neurite growth at 48–60 h later. As this culture duration precedes complete development of axonal versus dendritic polarity (Craig and Banker, 1994), we assessed overall neurite growth rather than axon growth in these cultures. The cortical neurons transfected with pre-miR-199a-3p showed significantly decreased neurite lengths, but transfection with pre-miR-21 had no effect (Fig. 5*A*,*B*). In contrast, transfection with anti-miR-21 decreased axon growth in the cortical neurons, while transfection with anti-miR199a-3p had no effect (Fig. 5*C*,*D*). These results indicate that miR-21 and miR-199a-3p levels influence the intrinsic growth ability of cortical neurons. Moreover, these findings suggest that similar to the injury-conditioned neurons, miR-21 is at saturating levels compared with miR-199a-3p in these embryonic cortical neurons.

Although the Houle lab used female rats for the SCI studies where miR-21 and miR-199a-3p levels were altered (Liu et al., 2012), we used male rats for the DRG cultures shown in Figures 1-4. Thus, we could not exclude the possibility of sexually dimorphic functional responses to miR-199a-3p and miR-21. Since the embryonic cortical neurons used here were from both male and female, we reasoned that dimorphic responses would generate two populations of neurons in the cortical cultures, ones whose neurite lengths were affected by the miR manipulations and a second where lengths were unaffected or affected in the opposite direction. To address this possibility, we evaluated the distribution of neurite lengths in the pre-miR and anti-miR transfected cortical neurons. Uni-modal distribution of neurite lengths were seen in pre-miR-21, pre-miR-199a-3p, anti-miR-21, and anti-miR-199a-3p transfected neurons comparable to controls (data not shown). This suggests that there miR-21 and miR-199a-3p have similar growth effects in male and female neurons.

### miR-21 overexpression supports neurite growth on non-permissive substrate

**Figure 6.**
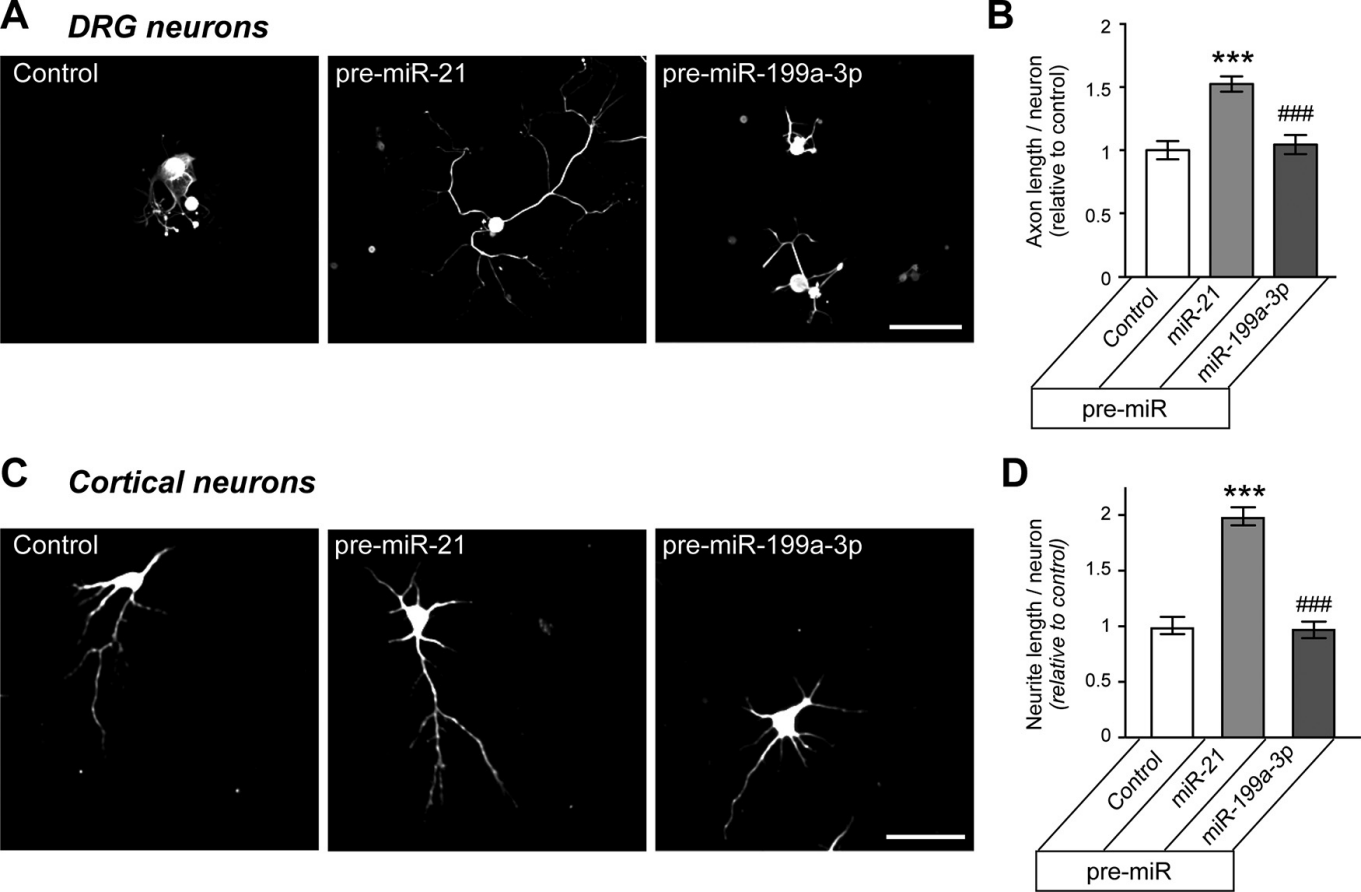
miR-21 overexpression supports neurite growth on non-permissive aggrecan substrate. ***A***, ***B***, Representative immunofluorescent images for NF in adult DRG cultures transfected with indicated pre-miRs versus scrambled RNA (control) and plated onto aggrecan (CSPG) are shown in ***A***. Total axon length per neuron is shown ***B*** as average ± SEM relative to control. ***C***, ***D***, Representative immunofluorescent images for NF in E18 cortical neuron cultures transfected with indicated pre-miRs versus scrambled RNA and plated onto aggrecan (CSPG) are shown in ***C***. Total axon length per neuron is shown ***D*** as average ± SEM relative to control (*n* ≥ 95 over each over 3 experimental replicates for ***B***, ***D***; ***p ≤ 0.005 versus control and ##*p* ≤ 0.01 and ###*p* ≤ 0.005 versus miR-21 by one-way ANOVA with pair-wise comparison and Tukey’s *post hoc* tests). Scale bar: 100 μm (***A***) and 50 μm (***C***).

Genetic deletion of PTEN was shown to facilitate axon regeneration in the non-permissive environment of the injured CNS (Park et al., 2008; Liu et al., 2010). Thus, we asked whether the growth promoting effects of increasing miR-21 or miR-199a-3p levels affects axon growth on the non-permissive CSPG aggrecan. Naïve adult DRG neurons transfected with pre-miR-21 showed significantly increased axon growth on the aggrecan compared with scrambled control RNA and pre-miR-199a-3p transfected neurons (Fig. 6*A*,*B*). Embryonic cortical neurons transfected with pre-miR-21 similarly showed significantly increased neurite growth on aggrecan compared the scrambled control RNA and pre-miR-199a-3p transfected cultures (Fig. 6*C*,*D*). Taken together, these data suggest that increased PTEN activity contributes to neurite growth inhibition by CSPGs, with miR-21 likely able to overcome this effect by decreasing PTEN abundance.

### Exogenous expression of miR-resistant mTOR and PTEN protein rescues miR-mediated axon growth effects

**Figure 7.**
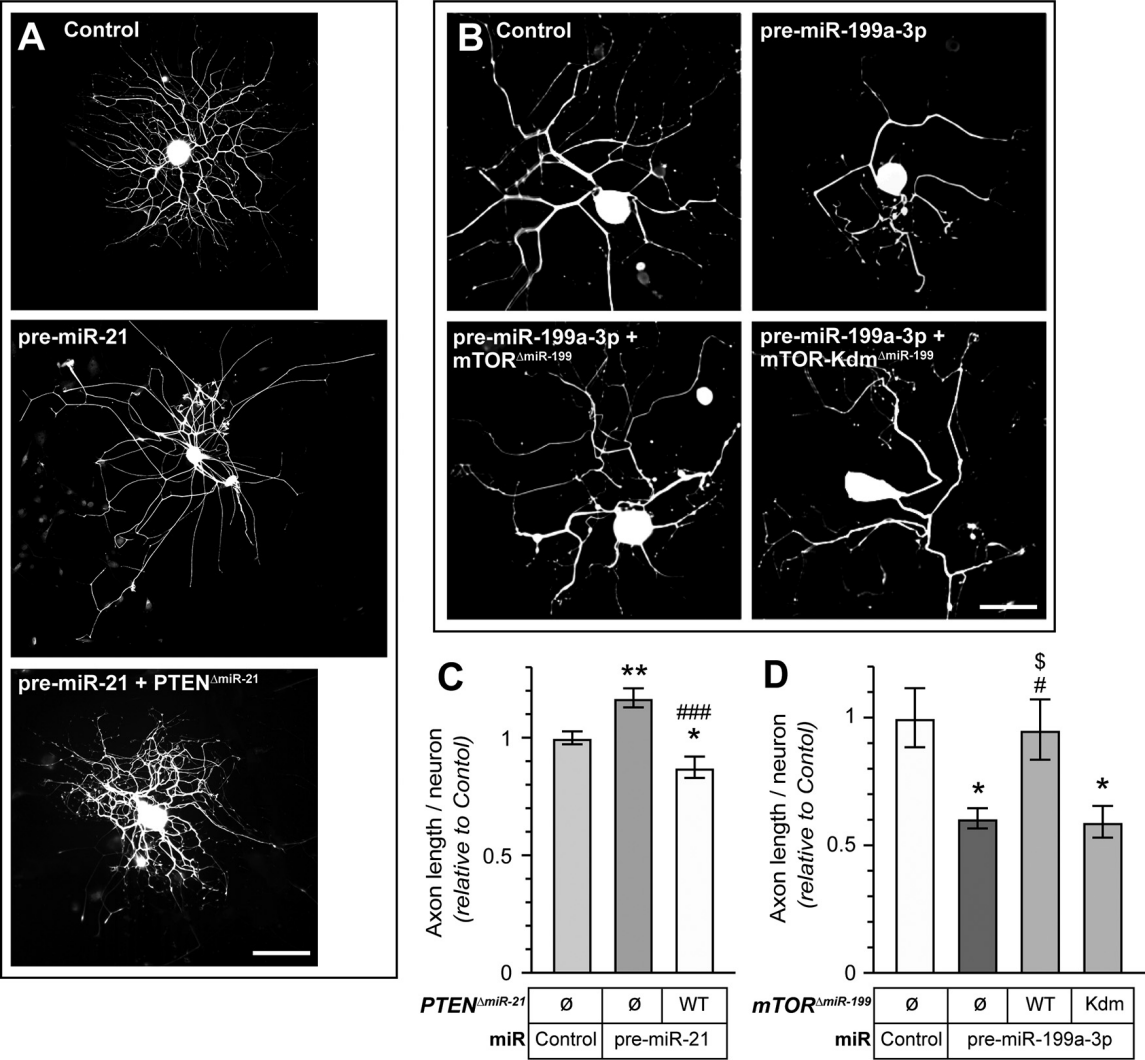
Exogenous *mTor* and *Pten* overcome the miR-199a-3p and miR-21 axon growth alterations. ***A***, ***B***, Representative immunofluorescent images for NF in adult DRG neurons co-transfected with indicated pre-miRs or scrambled RNA (control) plus expression constructs for plasmids encoding flag-tagged PTEN or mTOR with deletion of the predicted miR-199a-3p and miR-21 target sequences, respective (Pten^△miR-21^ and mTor^△miR-199^). Co-transfection with plasmids lacking the PTEN and mTOR coding sequences were used as control for ***A***, ***B***. miR-199a-3p target site deleted kinase-dead mutant mTOR with deletion of predicted miR-199a-3p target sequence (mTOR-Kdm^△miR-199^) was used in ***B***. ***C***, ***D***, Total axon length per neuron for DRG neurons transfected as in ***A***, ***B*** are shown as average ± SEM relative to control (*N* ≥ 50 neurons over 3 experimental replicates; *p ≤ 0.05 and **p ≤ 0.01 vs control, #*p* ≤ 0.05 and ###*p* ≤ 0.005 vs miR-21, and $*p* ≤ 0.05 vs mTOR-Kdm by one-way ANOVA with pair-wise comparison and Tukey’s *post hoc* tests). Scale bars: 100 μm (***A***, ***B***).

The data above raise the possibility that the balance of PTEN versus mTOR levels contribute to axon growth promotion and attenuation by miR-21 and miR-199a-3p, respectively. However, these experiments did not rule out contributions of other mRNA targets for the effects of miR-21 and miR-199a-3p on axon growth. To directly test whether modulation of *Pten* and *mTor* mRNA levels by these two miRs determines their effects on neurite growth, we asked whether co-transfection with miR-resistant *Pten* and *mTor* mRNAs could reverse the outcomes of miR-21 and miR-199a-3p over expression. For this, we generated PTEN and mTOR expression constructs with the predicted miR-21 and miR-199a-3p recognition sites deleted from the *Pten* and *mTor* mRNA 3′ UTRs (Pten^ΔmiR-21^ and mTor^ΔmiR-199^, respectively). Co-transfecting Pten^ΔmiR-21^ with pre-miR-21 in adult DRG neurons significantly attenuated the axon growth promotion effects seen with elevated miR-21 levels (Fig. 7*A*,*C*). Similarly, co-transfecting mTor^ΔmiR-199^ with pre-miR-199a-3p in adult DRG neurons significantly reversed the axon growth attenuation seen with elevated miR-199a-3p levels (Fig. 7*B*,*D*). Considering that elevation of mTOR activity has been linked to axon growth promotion rather than *mTor* mRNA levels, we asked whether mTOR kinase activity is required to rescue effects of miR-199a-3p overexpression. Thus, we generated a kinase dead, miR-199a-3p-resistant mTor expression construct (mTor-Kdm^ΔmiR-199^). In contrast to the wild-type miR-resistant mTOR, expression of mTor-Kdm^ΔmiR-199^ did not mitigate effects of pre-miR-199a-3p expression (Fig. 7*B*,*D*). Although we cannot completely rule out effects of other miR-21 and miR-199a-3p mRNA targets, our results indicate that depletion of *Pten* mRNA contributes to neurite growth promotion by miR-21 and depletion of *mTor* mRNA contributes to neurite growth attenuation by miR-199a-3p.

### Levels of miR-21 and miR-199a-3p affect axonal protein synthesis in DRG neurons

**Figure 8.**
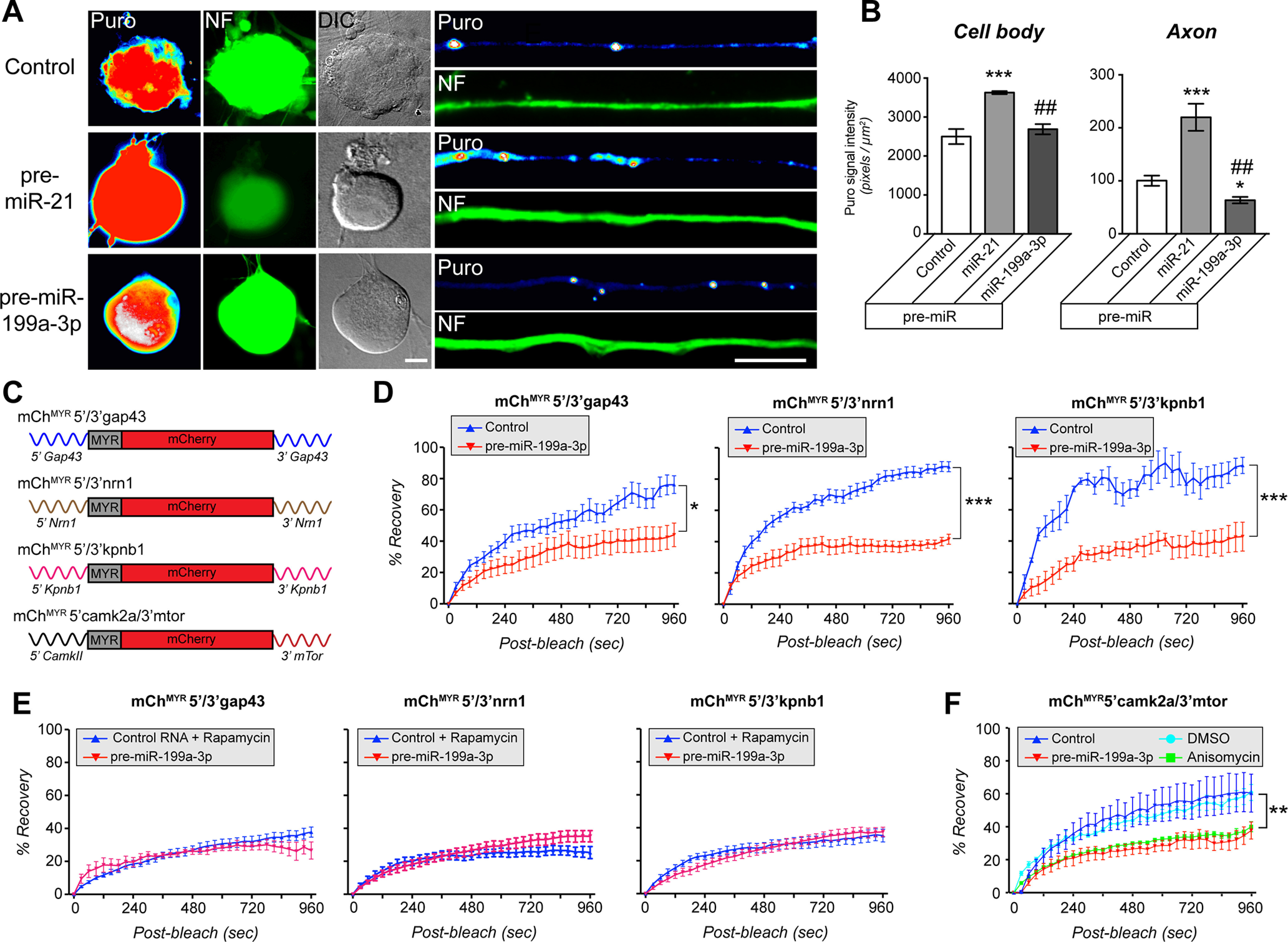
miR-199a-3p and miR-21 modulate neuronal protein synthesis. ***A***, ***B***, Representative fluorescent images for puromycin (Puro) incorporation in DRG neurons transfected with indicated pre-miRs or scrambled RNA (control) and treated with OPP are shown for cell bodies (left three columns) distal axons (right column). OPP incorporation was detected by labeling with Alexa Fluor 594 using the Click chemistry. ***B***, Average puromycin incorporation in cell bodies and axons (*N* ≥ 50 axons each over 3 experimental replicates; *p ≤ 0.001, ***p ≤ 0.001 vs control and ##*p* ≤ 0.005 vs miR-21 by one-way ANOVA with pairwise comparison and Tukey’s HSD *post hoc* tests). Scale bars: 10 μm. ***C***, Schematics of intra-axonal translation reporter constructs used for FRAP experiments for panels ***D–F*** and Extended Data Figure 8-1. ***D***, ***E***, Quantitation of FRAP sequences for distal axons of DRGs expressing *mCh^MYR^5’/3’gap43*, *mCh^MYR^5’/3’nrn1*, or *mCh^MYR^5’/3’kpnb1* mRNAs that were cotransfected with pre-miR-199a-3p or scrambled RNA are shown. Axonal mCherry fluorescent recovery for each reporter was significantly attenuated by miR-199a-3p overexpression. Values shown as normalized average % recovery ± SEM; representative image sequences shown in Extended Data Figure 8-1. ***E***, FRAP analysis for *mCh^MYR^5’/3’gap43*, *mCh^MYR^5’/3’nrn1*, or *mCh^MYR^5’/3’kpnb1* mRNA expressing DRG neurons that were transfected with scrambled RNA and treated with rapamycin 2 h before FRAP show diminished fluorescence recovery comparable to miR-199a-3p overexpression. Values shown as normalized average % recovery ± SEM; representative image sequences shown in Extended Data Figure 8-1. ***F***, FRAP analyses for distal axons of *mCh^MYR^5’camkII/3’mtor* mRNA expressing adult DRG transfected with either pre-miR-199a-3p or scrambled RNA are shown. Data are shown as normalized average % recovery ± SEM and representative image sequences shown in Extended Data Figure 8-1. Scrambled RNA transfected neurons were also analyzed after treatment with anisomycin or vehicle control (DMSO), with the anisomycin-treated neurons indicating translation dependence for the recovery (*N* ≥ 13 axons over ≥ 3 experimental replicates for ***D–F***; *p ≤ 0.01, **p ≤ 0.005, ***p ≤ 0.001 by one-way ANOVA with pairwise comparison and Tukey’s HSD *post hoc* tests).

Increase in mTOR activity is well established to increase protein synthesis through downstream modifications in activity of translation factors (Switon et al., 2017). Notably, work in the CNS injury models where *PTEN* gene is deleted or PTEN protein is inhibited have largely focused on downstream activation of S6 kinase (S6K) or phosphorylation of ribosomal protein S6 (rpS6) to indicate increased mTOR activity, rather than activation of neuronal protein synthesis (Park et al., 2008; Liu et al., 2010; Ohtake et al., 2014; Urban et al., 2019; Walker et al., 2019). Moreover, a novel pharmacological approach for neurite growth modulators indicated that activity of S6K can impede rather than support neurite growth (Al-Ali et al., 2017). Thus, we asked whether depletion of *Pten* or *mTor* mRNAs by overexpression of miR-21 and miR-199a-3p impact neuronal protein synthesis. We initially used a puromycinylation assay to quantify translation of endogenous mRNAs in the DRG cultures. DRG cultures transfected with pre-miR-21 showed significantly higher puromycin incorporation in the neuronal cell bodies than control RNA or pre-miR-199a-3p transfected neurons (Fig. 8*A*,*B*). This increased puromycin incorporation also occurred in the axons of the pre-miR-21 transfected neurons, while the pre-miR-199a-3p transfected neurons showed modest but significantly decreased puromycin incorporation (Fig. 8*A*,*B*). These data indicate that elevation of miR-21 increases both overall and intra-axonal protein synthesis, while elevation of miR-199a-3p has the opposite effect.

10.1523/ENEURO.0155-21.2021.f8-1 Extended Data Figure 8-1 Representative FRAP image sequences for DRGs expressing *mCh^MYR^5’/3’gap43* (***A***), *mCh^MYR^5’/3’nrn1* (***B***), *mCh^MYR^5’/3’kpnb1* (***C***), and *mCh^MYR^5’camkII/3’mtor* (***D***) mRNAs plus pre-miR-199a-3p or scrambled RNA (control) are shown (for quantifications, see Fig. 8*D–F*). Boxes represent the photobleached ROI. Schematic for the translation reporter constructs used are shown above each representative image sequence. Scale bars: 10 μm. Download Figure 8-1, TIF file.

Previous studies have indicated that *mTor* mRNA can be translated in distal axons, both in cultured neurons and *in vivo* (Kye et al., 2014; Terenzio et al., 2018), so it is intriguing that pre-miR-199a-3p transfection reduced axonal puromycinylation. Thus, we asked whether pre-miR-199a-3p transfection would affect translation of axonal mRNAs whose protein products have been linked to axon regeneration. We focused specifically on *Importin β1* (*Kpnb1*), *Neuritin 1* (*Nrn1*), and *Growth-associated protein 43* (*Gap43*) mRNAs whose 5′ and 3′ UTRs have been shown to support axonal localization and translation of their mRNAs in DRG neurons using fluorescent reporters (Merianda et al., 2013; Yoo et al., 2013; Perry et al., 2016; Sahoo et al., 2018, 2020; Terenzio et al., 2018). We used a FRAP assay in DRG cultures co-expressing pre-miR-199a-3p plus mCherry^MYR^ (mCh^MYR^) reporters with the 5′ and 3′ UTRs of *Kpnb1*, *Nrn1*, or *Gap43* mRNAs (mCh^MYR^5’/3’gap43, mCh^MYR^5’/3’kpnb1, and mCh^MYR^5’/3’nrn1; Fig. 8*C*). Axonal recovery after photobleaching in transfected DRGs was previously shown to be translation-dependent for each of these reporters (Sahoo et al., 2018, 2020). Expression of pre-miR-199a-3p significantly decreased recovery of photobleached mCh^MYR^5’/3’gap43, mCh^MYR^5’/3’kpnb1, and mCh^MYR^5’/3’nrn1 in distal axons of the cultured DRG neurons (Fig. 8*D*). Moreover, in control RNA transfected cultures, axon pretreatment with the mTOR inhibitor rapamycin before photobleaching decreased recovery of mCh^MYR^5’/3’gap43, mCh^MYR^5’/3’kpnb1, and mCh^MYR^5’/3’nrn1 comparable to the recovery seen in miR-199a-3p overexpressing neurons (Fig. 8*E*). We next asked whether overexpression of miR-199a-3p would affect intra-axonal translation of *mTor* mRNA. For this we generated an mCh^MYR^ reporter with the 3′ UTR of *mTor* mRNA containing the predicted miR-199a-3p target sequence and the 5′ UTR of *Camk2a* mRNA (mCh^MYR^5’camk2a/3’mtor; Fig. 8*C*; Extended Data Fig. 8-1*A–C*). The *mTor* 3′ UTR is sufficient for axonal localization (Terenzio et al., 2018), while the *Camk2a* supports translation in axons but has no axonal localizing activity (Merianda et al., 2013). FRAP for the mCh^MYR^5’camk2a/3’mtor transfected DRG axons showed that cotransfection with pre-miR-199a-3p reduced fluorescence recovery comparable to what was seen with the protein synthesis inhibitor anisomycin (Fig. 8*F*; Extended Data Fig. 8-1*D*). Taken together, these data indicate that manipulation of neuronal PTEN and mTOR activity through posttranscriptional mechanisms change both overall and intra-axonal protein synthesis.

## Discussion

Here, we show that miR-21 promotes axon growth from adult DRG neurons and neurite growth from embryonic cortical neurons, while miR-199a-3p does the opposite. Consistent with the increased axonal growth seen after peripheral nerve injury conditioning, miR-21 levels are increased in injury-conditioned DRG neurons while levels of miR-199a-3p decreased. Importantly, we show that miR-21 binds to and depletes *Pten* mRNA and miR-199a-3p binds to and depletes *mTor* mRNA in adult neurons, with commensurate decreases in the proteins encoded by these mRNAs. Furthermore, the growth promoting effects of miR-21 and inhibiting effects of miR-199a-3p are completely reversed by introduction of *Pten* and *mTor* mRNAs lacking the corresponding target sites for these miRs in their 3′ UTRs. While these miRs undoubtedly have many mRNA targets, including several with roles in neurite growth that have been recently published (Li et al., 2020; Liu et al., 2020; Song et al., 2020; Laliberte et al., 2021; Nakashima et al., 2021), our findings emphasize that depletion of PTEN through increased miR-21 increases axon growth and depletion of mTOR through increased miR-199a-3p decreases axon growth.

Several studies have linked alterations in miR levels, including miR-21, to promotion of axon growth and regeneration in the PNS and CNS. For example, co-transfection of miR-21 and miR-338 into rat spinal cord neurons increased regeneration after sciatic nerve injury (Wang et al., 2016). Lopez-Leal et al. (2020) recently reported that cultures of denervated Schwann cells secrete miR-21 in exosomes, and those exosomes decrease PTEN levels and increase axon outgrowth when applied to DRG explant cultures (Lopez-Leal et al., 2020). Exosomes isolated from mesenchymal stem cells and differentiated PC12 cells that contain miR-21 and miR-19b were also shown to increase recovery from SCI in rodents (Xu et al., 2019). Further exercise-induced miR-21 in the injured spinal cord is also packaged into exosomes (Li et al., 2020). However, there are other reports that increased miR-21 in the injured spinal cord correlate with poor recovery (Chung et al., 2020), and knock-down of miR-21 improved recovery after SCI with alleviation of peripheral inflammatory cytokine expression and BDNF elevation in the injured spinal cord (Xie et al., 2018). The basis of these data that conflict with previous work showing elevated miR-21 with exercise after SCI is not clear, and our data unequivocally show that increasing neuronal miR-21 increases axon growth, including growth on the non-permissive CSPG substrate aggrecan.

Our data indicate that increased miR-199a-3p expression reduces *mTor* mRNA translation in axons and results in decreased translation of axonal mRNAs encoding proteins linked to an increase in PNS axon regeneration. Although it is not clear whether miR-199a-3p localizes into injured spinal cord axons as we see with the DRG cultures here, our data suggest that the SCI-induced increase miR-199a-3p reported by Liu et al. (2012) could decrease capacity for axon regeneration in those animals. Similarly, the reported exercise-induced increase in miR-21 and decrease in miR-199a-3p after SCI (Liu et al., 2012) could shift the injured neurons to a fate favorable for axon regeneration. Consistent with this, exercise of rats post-SCI has been shown to increase the number of sensory axons regenerating into a peripheral nerve grafted into the transected spinal cord (Sachdeva et al., 2016). Further, work from Li et al. (2020) recently showed that exercise-associated miR-21 increase correlates with increased functional recovery from SCI with the elevated miR-21 targeting programmed cell death protein 4 (PCD4; Li et al., 2020). In some paradigms, exercise has also been shown to increase axon regeneration after peripheral nerve injury (Sabatier and English, 2015; Cannoy et al., 2016). Furthermore, DRG neurons cultured from adult rats that have undergone voluntary exercise showed increased *in vitro* axon growth that statistically correlated with the distance the animals had run (Molteni et al., 2004). These data suggest that the increase in miR-21 and decrease in miR-199a-3p detected in the injury-conditioned DRG neurons here could indeed contribute to ability faster axon extension seen in those neurons. However, it should be noted that miR-21 and miR-199a-3p do not always show reciprocal regulation as seen here, since both miRs were decreased in DRGs from diabetic mice (Cheng et al., 2015).

Many studies have now shown that the PTEN/mTOR pathway can alter growth potential of neurons, with increased mTOR activity supporting axon regeneration in both the CNS and PNS (Park et al., 2008; Abe et al., 2010; Liu et al., 2010; Sun et al., 2011). Phosphorylation of S6K and RP S6 have often been taken as evidence for increased mTOR activity and the resulting increased mRNA translation in these axon injury model systems (Park et al., 2008; Abe et al., 2010; Liu et al., 2010; Sun et al., 2011; Urban et al., 2019). Notably, although phosphorylated RP S6 tends to be associated with translationally active ribosomes, the phosphorylation status of RP S6 does not always reflect a change in protein synthesis (Biever et al., 2015). Recent work using S6K inhibitors indicates that S6K activation can attenuate CNS axon regeneration (Al-Ali et al., 2017). Our data show that altering the balance of PTEN/mTOR levels in neurons does indeed alter neuronal protein synthesis, including translation through *Kpnb1*, *Nrn1*, and *Gap43* mRNA UTRs in axons. This is consistent with recent work pointing to loss of axonal protein synthesis when *mTor* mRNA’s localization into sciatic nerve axons was attenuated through deletion of its 3′ UTR (Terenzio et al., 2018; Sahoo et al., 2020).

The increase in miR-21 and decrease in miR-199a-3p levels in injury-conditioned neurons is consistent with previous work pointing to posttranscriptional regulation of axon growth from injury-conditioned DRG neurons (Smith and Skene, 1997; Twiss et al., 2000). Our data suggest that miR-21 is at saturating levels in both the injury-conditioned DRG neurons and embryonic cortical neurons, as miR-21 overexpression did not further increase neurite growth in either preparation. However, overexpression of miR-199a-3p was able to decrease neurite growth in both neuronal populations indicating that depleting *mTor* mRNA from these neurons can overcome effects of saturating miR-21 levels. PNS neurons have been hypothesized to switch to an embryonic gene expression program that supports axon growth after injury, and recent profiling of ribosome bound mRNAs in corticospinal neurons after SCI indicates that these CNS neurons similarly shift to an embryonic gene expression program, with regeneration promoting stem cell grafts causing sustained expression of the growth-associated genes (Poplawski et al., 2020). Our observations that miR-21 is saturated for growth on permissive substrates in both the injury-conditioned adult DRG and embryonic cortical neurons raise the possibility that posttranscriptional regulation of gene expression may also shift toward a more embryonic state to support growth programs through modulation of PTEN to mTOR pathway. PTEN can modify activity of other downstream targets beyond mTOR, and increased neurite growth in murine DRG cultures after inhibition or depletion of PTEN was shown to be insensitive to mTOR inhibition with rapamycin (Cheng et al., 2015). However, rapamycin-resistant mTOR activity was more recently reported to support regeneration of injury-conditioned DRG axons, both in vivo after SCI and in culture (Chen et al., 2016). Consistent with this, localized mTOR activity supports regeneration of injured PNS axons through its translation promoting activity (Terenzio et al., 2018; Sahoo et al., 2020).

The Court lab recently reported that transition of Schwann cells to a repair phenotype results in secretion of exosomes containing miR-21. Similar to our data, they reported increased axonal growth with depletion of *Pten* mRNA from in cultured DRG neurons exposed to these Schwann cell-derived exosomes (Lopez-Leal et al., 2020). Since the DRG cultures used here contain Schwann cells that have lost contact with axons, we cannot exclude that some of the changes in miR-21 levels derive from Schwann cells. Nonetheless, our data emphasize a direct effect of miR-21 on neuronal PTEN expression and we see that endogenous miR-21 and miR-199a-3p localize into axons of the cultured DRG neurons.

miRs have been detected in axons of many different neuronal populations with numerous functional effects seen with overexpression or depletion of these miRs. To our knowledge, this is the first report of axonal miRs directly targeting *Pten* and *mTor* mRNAs that subsequently regulate translation of other mRNAs, including mRNAs localizing into growing axons. Interestingly, our manipulations of the PTEN/mTOR pathway and resulting effects on protein synthesis by overexpression and inhibition of miR21 and miR-199a-3p selectively affected axon elongation rather than axon branching. Transcriptional programs associated with sprouting or branching of sensory neurons have been reported for both in vivo and cultured neurons (Harrison et al., 2015; Lemaitre et al., 2020). In contrast to these findings, our data emphasize that the elongating axon growth regulated by the balance of miR-21 and miR-199a-3p levels is impacted by direct posttranscriptional regulation of *Pten* and *mTor* mRNAs.

## Synthesis

Reviewing Editor: Eran Perlson, Tel Aviv University Decisions are customarily a result of the Reviewing Editor and the peer reviewers coming together and discussing their recommendations until a consensus is reached. When revisions are invited, a fact-based synthesis statement explaining their decision and outlining what is needed to prepare a revision will be listed below. The following reviewer(s) agreed to reveal their identity: Angelo Lepore, Douglas Zochodne.

This is an important, comprehensive and very well-done set of studies exploring the role of two miR’s on the axon growth capacity of adult DRG and embryonic cortical neurons. Specifically, the authors examined how miR-21 and miR-199a-3p regulate Pten and mTor mRNA (and eventual protein expression), respectively; PTEN and mTor are two centrally important regulators of axon growth potential, including in the context of nervous system damage. The authors present a large amount of high-quality data. In addition to the excellent studies presented in this paper, the manuscript is also very well written.

Still, before publication, the authors need to clarify and address the following:

1. The authors used only male rats for the primary DRG culture experiments, while both females and males were used for the embryonic cortical neuron studies. Is there any reason (i.e. data in the field and/or from their own lab) to think that there could be sexually dimorphic roles of these two particular miR’s (and/or other miR’s) on the axon growth response of DRG neurons and/or other neuronal populations?

2. There are a few typos in the manuscript that needs to be fixed. For example, the third sentence of the Results section says, “Moreover, the predictions that miR-21 would directly neuronal Pten mRNA and miR-199a-3p would directly neuronal mTor mRNA were not directly tested.” Fig2 F/J switch blots? Fig 8F mixed graphs?

3.It is very likely that these two miRNAs have very many more targets than simply PTEN or mTOR. two convenient targets identified. Other candidates might have to be considered and discussed in the discussion section.

4. The presence of miR-21 and miR-199a-3p in adult DRGs has already been reported (not cited here) in an examination of experimental diabetic models (Cheng et al, J NP Exp Neurol 74: 804,2015). In that report, both were downregulated by diabetes which might make interpreting them as having primarily opposite actions an oversimplification. Please discuss this.

5. The work assumes that PTEN and mTOR are closely coupled (at the exclusion of a lot of other related interactions). This indeed was seen in the CNS by Park et al, but in PNS work (not cited) Christie et al (J Neurosci. 2010 Jul 7;30(27):9306) did not identify a tight linkage, also shown by others in the PNS. In the current work, the authors do not make a convincing case that their two approaches would have to be linked. Hence consideration of this literature and skepticism of an essential link would improve the overall findings. The authors have shown that both pathways are important for growth but do not indicate that one has to follow the other. Full discussion over this is required as it’s an interesting issue in PTEN signaling.

6. Unclear if the authors use n=3 or 4 as the actual n number when applying statistics to the in vitro work. Please clarify the n and statistics tests.

7. Note that declines in PTEN after axotomy are not necessarily the case, weakening the argument that it’s the mir-21 that is responsible. The authors should discuss what other miRNAs can target PTEN and how are they differentially regulated after axotomy?
