# Supplementary figures and images for: MicroRNAs 21 and 199a-3p Regulate Axon Growth Potential through Modulation of Pten and mTor mRNAs
Source: eNeuro. 2021 Aug 10;8(4):ENEURO.0155-21.2021. doi: 10.1523/ENEURO.0155-21.2021 (PMC8362682; doi:10.1523/ENEURO.0155-21.2021)

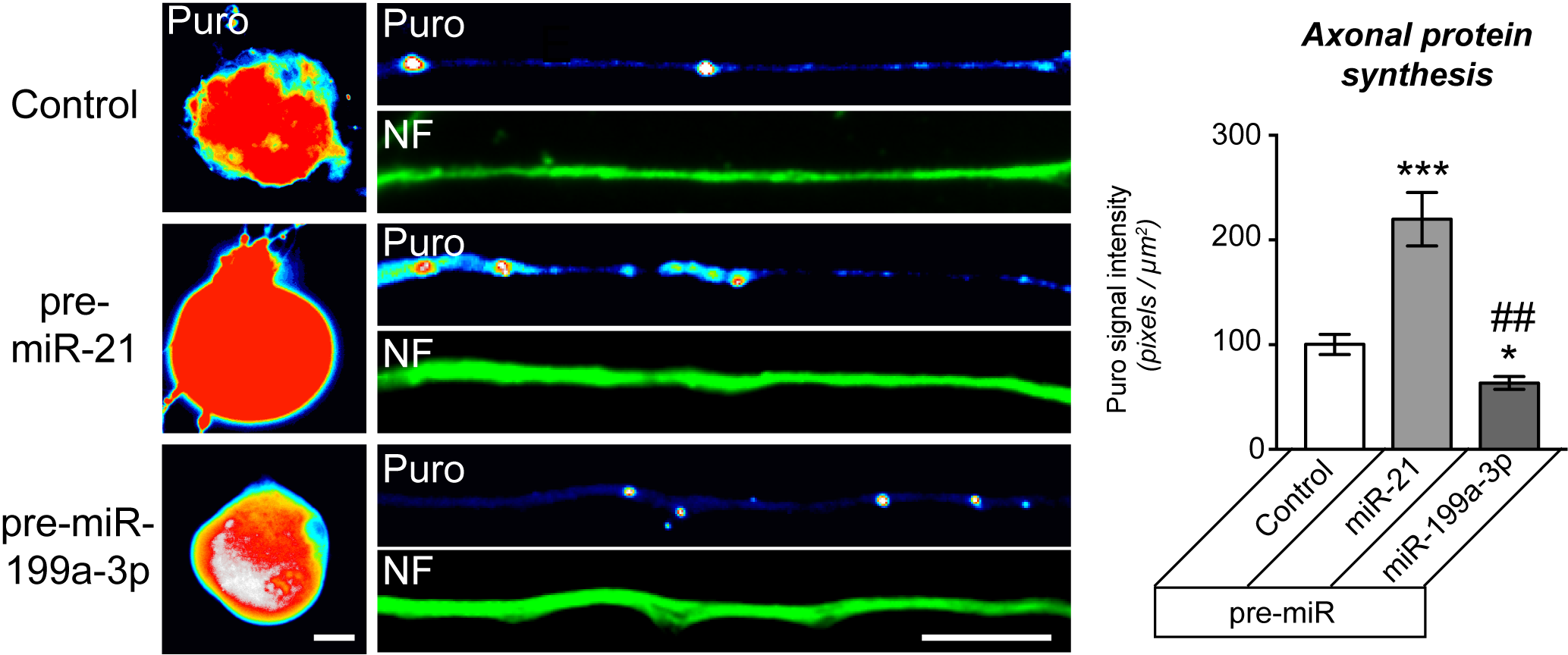

Supplement: Extended Data Figure 8-1 — Representative FRAP image sequences for DRGs expressing mChMYR5’/3’gap43 (A), mChMYR5’/3’nrn1 (B), mChMYR5’/3’kpnb1 (C), and mChMYR5’camkII/3’mtor (D) mRNAs plus pre-miR-199a-3p or scrambled RNA (control) are shown (for quantifications, see Fig. 8D–F). Boxes represent the photobleached ROI. Schematic for the translation reporter constructs used are shown above each representative image sequence. Scale bars: 10 μm. Download Figure 8-1, TIF file. [file enu-eN-NWR-0155-21-s03.tif]
